# Supplementary material for: MetaRibo-Seq measures translation in microbiomes
Source: Nat Commun. 2020 Jun 29;11:3268. doi: 10.1038/s41467-020-17081-z (PMC7324362; doi:10.1038/s41467-020-17081-z)
Supplement: Supplementary file 10 — Supplementary Data 7 [file 41467_2020_17081_MOESM10_ESM.zip › File2/Confidence_High_Taxonomy/305426_out.krona.html]

Javascript must be enabled to view this page.

members
magnitude
magnitudeUnassigned
count
unassigned
taxon
rank

305426\_out

256

superkingdom
2
256

255
976
phylum

class
1
117743

1
200644
order


SRS142503\_contig\_number\_39265
species
1
1950957

254
200643
class

254
171549
order

family
171550
254

239759
254
16

SRS012273\_contig\_number\_23512SRS012969\_contig\_number\_2804SRS016335\_contig\_number\_45123SRS016381\_contig\_number\_2914SRS018351\_contig\_number\_3177SRS018984\_contig\_number\_2804SRS047044\_contig\_number\_contig-100\_2087.90106SRS048164\_contig\_number\_25155SRS049446\_contig\_number\_5926SRS050299\_contig\_number\_11444SRS050925\_contig\_number\_5724SRS074586\_contig\_number\_5513SRS076756\_contig\_number\_contig-100\_913.115853SRS077086\_contig\_number\_11080SRS144753\_contig\_number\_10997SRS893380\_contig\_number\_1086
genus

2
1946009

SRS019445\_contig\_number\_4588SRS142890\_contig\_number\_6764
species

1263035
4
species

SRS011084\_contig\_number\_2192SRS024009\_contig\_number\_8036SRS142923\_contig\_number\_11615SRS148159\_contig\_number\_52391


SRS015782\_contig\_number\_27911SRS017622\_contig\_number\_265SRS147425\_contig\_number\_9034
species
2292910
3

328814
82

SRS011239\_contig\_number\_255SRS011405\_contig\_number\_17041SRS012849\_contig\_number\_3463SRS013098\_contig\_number\_27820SRS013800\_contig\_number\_17695SRS014235\_contig\_number\_38721SRS014613\_contig\_number\_12015SRS015579\_contig\_number\_14550SRS015890\_contig\_number\_14562SRS016203\_contig\_number\_5332SRS016267\_contig\_number\_7942SRS017307\_contig\_number\_contig-100\_839.144694SRS017701\_contig\_number\_contig-100\_672.132455SRS017859\_contig\_number\_4806SRS018427\_contig\_number\_32080SRS018836\_contig\_number\_23016SRS019787\_contig\_number\_9485SRS019808\_contig\_number\_contig-100\_1149.117657SRS019910\_contig\_number\_13360SRS020271\_contig\_number\_3762SRS020328\_contig\_number\_4960SRS020394\_contig\_number\_12840SRS022137\_contig\_number\_11341SRS023715\_contig\_number\_11851SRS045195\_contig\_number\_2708SRS045645\_contig\_number\_904SRS045713\_contig\_number\_11368SRS047433\_contig\_number\_21837SRS049896\_contig\_number\_33582SRS049995\_contig\_number\_1551SRS052027\_contig\_number\_contig-100\_754.85752SRS054905\_contig\_number\_14569SRS055982\_contig\_number\_12166SRS058723\_contig\_number\_7297SRS058770\_contig\_number\_24041SRS062654\_contig\_number\_27036SRS063040\_contig\_number\_45150SRS063370\_contig\_number\_12781SRS064276\_contig\_number\_16369SRS065176\_contig\_number\_11517SRS077024\_contig\_number\_9881SRS077294\_contig\_number\_12664SRS077335\_contig\_number\_8017SRS078419\_contig\_number\_12779SRS097920\_contig\_number\_28311SRS097958\_contig\_number\_16139SRS098073\_contig\_number\_7420SRS098644\_contig\_number\_14663SRS098881\_contig\_number\_contig-100\_1638.158277SRS1041033\_contig\_number\_24382SRS1041037\_contig\_number\_15198SRS1041038\_contig\_number\_16906SRS1041092\_contig\_number\_8887SRS1041095\_contig\_number\_1725SRS1041133\_contig\_number\_28017SRS1041136\_contig\_number\_20028SRS1041137\_contig\_number\_12052SRS1041138\_contig\_number\_3708SRS1041141\_contig\_number\_5336SRS1041143\_contig\_number\_6668SRS1041147\_contig\_number\_1959SRS104400\_contig\_number\_24489SRS104912\_contig\_number\_13944SRS104975\_contig\_number\_1829SRS1055038\_contig\_number\_contig-100\_1524.40178SRS143085\_contig\_number\_2731SRS143417\_contig\_number\_17211SRS143895\_contig\_number\_5020SRS144297\_contig\_number\_14067SRS144603\_contig\_number\_15692SRS144714\_contig\_number\_25543SRS146764\_contig\_number\_32852SRS146812\_contig\_number\_44005SRS148091\_contig\_number\_13327SRS148424\_contig\_number\_7421SRS148874\_contig\_number\_2608SRS148970\_contig\_number\_2413SRS149181\_contig\_number\_29586SRS149325\_contig\_number\_15737SRS893252\_contig\_number\_19682SRS971275\_contig\_number\_7105SRS971276\_contig\_number\_5466
species

species

SRS023526\_contig\_number\_37112SRS049959\_contig\_number\_29252SRS062427\_contig\_number\_9166SRS893383\_contig\_number\_12144
4
1288121

1
2291998
species

SRS015663\_contig\_number\_21257

4
28117
species

SRS018623\_contig\_number\_1185SRS053649\_contig\_number\_10322SRS077849\_contig\_number\_5580SRS144135\_contig\_number\_34631


SRS055017\_contig\_number\_18895SRS893341\_contig\_number\_2351SRS893358\_contig\_number\_8844SRS893378\_contig\_number\_17312
species
4
1262697


SRS1041036\_contig\_number\_5043
species
1
2364787

species

SRS020508\_contig\_number\_contig-100\_3423.3424SRS078242\_contig\_number\_10834SRS098881\_contig\_number\_19067
1816676
3

4
214856

SRS064757\_contig\_number\_21440SRS103987\_contig\_number\_20613SRS1041129\_contig\_number\_3971SRS145497\_contig\_number\_10594
species

59
1118061

SRS011084\_contig\_number\_21902SRS011239\_contig\_number\_23181SRS014855\_contig\_number\_3244SRS015663\_contig\_number\_36513SRS015854\_contig\_number\_5205SRS015960\_contig\_number\_6294SRS016095\_contig\_number\_contig-100\_1244.164381SRS017103\_contig\_number\_2915SRS017191\_contig\_number\_19836SRS018351\_contig\_number\_11017SRS018541\_contig\_number\_2826SRS019445\_contig\_number\_33594SRS020394\_contig\_number\_6336SRS021948\_contig\_number\_22009SRS023971\_contig\_number\_3949SRS043701\_contig\_number\_14669SRS045645\_contig\_number\_521SRS049402\_contig\_number\_9414SRS049773\_contig\_number\_2802SRS049773\_contig\_number\_28388SRS049959\_contig\_number\_16822SRS049995\_contig\_number\_15968SRS050925\_contig\_number\_35442SRS053649\_contig\_number\_14961SRS054352\_contig\_number\_11198SRS054352\_contig\_number\_13145SRS054905\_contig\_number\_23974SRS056259\_contig\_number\_14578SRS056519\_contig\_number\_13341SRS057480\_contig\_number\_contig-100\_174.80834SRS065504\_contig\_number\_27712SRS075078\_contig\_number\_12964SRS075773\_contig\_number\_19401SRS075984\_contig\_number\_21810SRS076929\_contig\_number\_contig-100\_1041.148867SRS077194\_contig\_number\_14112SRS077231\_contig\_number\_10576SRS077294\_contig\_number\_contig-100\_1277.114344SRS077552\_contig\_number\_24339SRS077641\_contig\_number\_18308SRS097958\_contig\_number\_24678SRS098061\_contig\_number\_3598SRS098644\_contig\_number\_30554SRS098717\_contig\_number\_16253SRS098881\_contig\_number\_14285SRS101376\_contig\_number\_2133SRS101433\_contig\_number\_10445SRS1041136\_contig\_number\_9461SRS140492\_contig\_number\_21596SRS143372\_contig\_number\_10523SRS143895\_contig\_number\_6336SRS143991\_contig\_number\_28677SRS144135\_contig\_number\_33884SRS144297\_contig\_number\_contig-100\_1650.104163SRS144537\_contig\_number\_22080SRS147271\_contig\_number\_11794SRS148319\_contig\_number\_6362SRS148817\_contig\_number\_6158SRS149075\_contig\_number\_11158
species


SRS1055056\_contig\_number\_contig-100\_1087.73285
species
1
1465754

66
1896969
species

SRS011134\_contig\_number\_3382SRS013382\_contig\_number\_contig-100\_1242.1243SRS013521\_contig\_number\_6736SRS013638\_contig\_number\_18053SRS013940\_contig\_number\_18776SRS014313\_contig\_number\_4116SRS014415\_contig\_number\_6SRS015694\_contig\_number\_13701SRS016018\_contig\_number\_2334SRS016753\_contig\_number\_8326SRS016954\_contig\_number\_21038SRS017433\_contig\_number\_16696SRS017821\_contig\_number\_27468SRS017916\_contig\_number\_731SRS018133\_contig\_number\_7183SRS018541\_contig\_number\_1210SRS019161\_contig\_number\_21852SRS019178\_contig\_number\_4405SRS019685\_contig\_number\_26354SRS019968\_contig\_number\_14506SRS021219\_contig\_number\_6622SRS022609\_contig\_number\_47490SRS024331\_contig\_number\_35254SRS024492\_contig\_number\_18949SRS042628\_contig\_number\_25488SRS043768\_contig\_number\_16191SRS043841\_contig\_number\_607SRS044535\_contig\_number\_16473SRS045739\_contig\_number\_10477SRS046068\_contig\_number\_1645SRS046369\_contig\_number\_10577SRS049959\_contig\_number\_21630SRS050026\_contig\_number\_11177SRS050941\_contig\_number\_15577SRS051882\_contig\_number\_9361SRS054059\_contig\_number\_4702SRS063190\_contig\_number\_contig-100\_1751.86128SRS063985\_contig\_number\_7822SRS074670\_contig\_number\_3462SRS075078\_contig\_number\_12221SRS076976\_contig\_number\_4767SRS077231\_contig\_number\_22903SRS077502\_contig\_number\_17331SRS077589\_contig\_number\_2480SRS078665\_contig\_number\_4295SRS1041032\_contig\_number\_8643SRS1041039\_contig\_number\_19987SRS1041118\_contig\_number\_17491SRS105153\_contig\_number\_5832SRS1054928\_contig\_number\_7488SRS1055022\_contig\_number\_800SRS1055099\_contig\_number\_10898SRS140513\_contig\_number\_10059SRS143181\_contig\_number\_13865SRS143342\_contig\_number\_6007SRS144362\_contig\_number\_4630SRS144506\_contig\_number\_30519SRS144537\_contig\_number\_30159SRS144537\_contig\_number\_39074SRS145308\_contig\_number\_1229SRS148319\_contig\_number\_16841SRS150029\_contig\_number\_15263SRS893230\_contig\_number\_2334SRS893279\_contig\_number\_14801SRS893373\_contig\_number\_21380SRS893382\_contig\_number\_5926

1
1239
phylum

1
186801
class

order
186802
1

541000
1
family

genus
1
1263

1
1262952
species

SRS049773\_contig\_number\_16550
